# Lippes loop intrauterine device left in the uterus for 40 years as a rare cause of postmenopausal pelvic pain: a case report and review of the literature

**DOI:** 10.1186/s13256-023-03991-1

**Published:** 2023-09-02

**Authors:** Temesgen Tilahun, Asfaw Tadesse, Rut Oljira

**Affiliations:** 1 Department of Obstetrics & Gynecology, Institute of Health Sciences, Wollega University, P.O Box: 395, Nekemte, Oromia Ethiopia; 2 Department of Obstetrics & Gynecology, Nekemte Specialized Hospital, Oromia Regional Health Bureau, Nekemte, Ethiopia; 3 Department of Public Health, Institute of Health Sciences, Wollega University, Nekemte, Ethiopia

**Keywords:** Lippes loop, IUD, Retained, Contraception, Pelvic pain, Western Ethiopia

## Abstract

**Background:**

Intrauterine devices are a widely used method of contraception worldwide. These devices are reliable, cost-effective, long-acting, and reversible. Their placement in the uterus is usually simple and safe. Forgotten IUDs carry some complications and can adversely affect the health of women. Therefore, appropriate counseling during insertion and timely removal are crucial.

**Case summary:**

We present the case of retained Lippes loop IUD for 40 years in a 75-year-old postmenopausal patient from Western Ethiopia. The patient presented to the hospital with postmenopausal pelvic pain. The loop was removed with spongy forceps. The patient was discharged with analgesia and doxycycline twice a day for 3 days.

**Conclusion:**

Different works of the literature showed that retained Lippes loop IUD carries some complications. Our case was also presented with postmenopausal pelvic pain. Therefore, we recommend the removal of IUDs at their expiry date or menopause.

## Introduction

The first Lippes loop intrauterine device (IUD) was introduced in 1962. It was a plastic double "S" loop, a trapezoid-shaped IUD that closely fit around the contours of the uterine cavity, reducing the incidence of expulsion. This IUD was commonly used from the 1960s to the 1980s [1, 2]. Though it is safe to use, Lippes Loop IUD is no longer in use after Ortho Pharmaceutical Corporation stopped marketing this device citing economic reasons [2].

Lippes loop IUDs were intended for long-term use until menopause due to their implant nature. For this reason, they are often retained for years. Many patients present well into menopause still bearing a Lippes Loop either deliberately or forgotten [3]. However different literature have documented side effects & complications following long term use of IUDs [4].

Retained IUDs beyond the required time are related to numerous complications [5–10]. However, there are cases with no symptoms though kept for many years [1, 5]. There are controversies about whether to remove or conservatively managed dislocated or retained IUDs with no complaints [5, 7]. Here we presented a case of Lippes loop IUD retained for 40 years.

## Case presentation

This is a 75-year-old para 6 patient from Western Ethiopia who saw her last menses before 25 years. She had occasional pelvic pain for the last 3 years for which she was visiting different health facilities. Currently, she presented to Nekemte Specialized Hospital with exacerbation of lower abdominal pain of 3 weeks. She feels discomfort in her vagina but no protrusion of mass through her vagina. She has no history of fever, abdominal swelling, vaginal discharge, or bleeding. All her previous deliveries were normal vaginal deliveries. Upon enquiring about the history of family planning utilization; she reported that the intrauterine device was inserted before 40 years at a public hospital. Since then she had no history of gynecologic evaluation for a checkup. She had no history of gynecologic procedures, pelvic or abdominal surgery. The patient has no history of medical problems like diabetes mellitus, hypertension, cardiac or renal problems.

On examination, she was acutely sick-looking. Her vital signs were blood pressure (BP) = 120/80 mmHg, pulse rate (PR) = 82 beats per minute, respiratory rate (RR) = 18 breaths per minute, and temperature 37.1 °C. She had pink conjunctivae. Lymph glandular system, chest, and cardiovascular system were normal. On abdominal examination, there was no mass, organomegaly, area of tenderness, or signs of fluid collection. Inspection of external genitalia showed no vulvar mass or lesion. On speculum examination, there is a foreign body protruding through the cervix. However, there is no other cervical mass or lesion. On bimanual examination, the uterus is not enlarged and there was no adnexal mass or tenderness. On the integumentary system, she had no palmar pallor. On neurologic examination, she was oriented to time, person, and place. She had normal reflexes and no neurologic deficits.

**Fig. 1:**
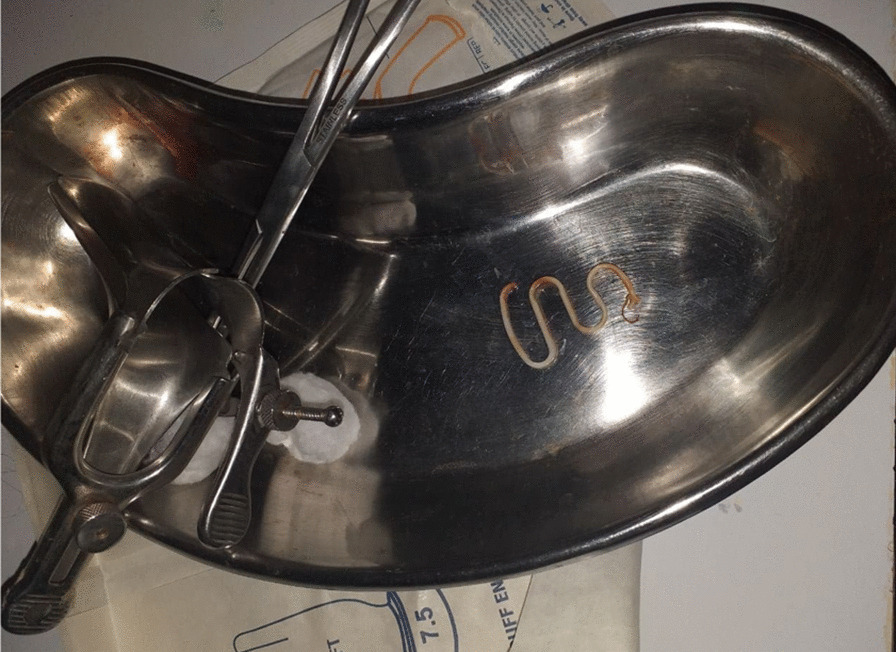
Lippes loop intrauterine device removed from a 75-year-old patient at Nekemte Specialized Hospital, 2021

On laboratory investigation, ultrasound examination was done by a radiologist and showed unremarkable pelvic findings. Urinalysis, complete blood count, and serum blood glucose level were normal. With the final diagnosis of postmenopausal pelvic pain secondary to retained intrauterine device, the patient was prepared and taken to the gynecology procedure room. On lithotomy position, the speculum was inserted and the intrauterine device was removed with spongy forceps (Fig. 1). The mother was observed for 4 h and discharged with analgesia and doxycycline 100 mg PO twice a day for three days.

## Discussion

Management of retained or dislocated IUDs is controversial [5]. Some authors stated that this IUD can be left in the uterine cavity for an indefinite amount of time [1, 9]. Other works of literature recommend removal [8]. Prolonged use of this device was common [11], however, it is associated with some problems such as uterine infection, uterine bleeding, uterine perforation, bowel perforation, and pelvic abscess [5–7]. In postmenopausal women, it is related to chronic pelvic pain, pyometra, and abnormal uterine bleeding [7, 12, 13].

In this case, the patient was frequently visiting health centers and clinics for recurrent pelvic pain. She was treated with different analgesics. The pain might result from embedment of the uterine wall by the device, recurrent pelvic inflammatory disease, and chronic inflammatory response of the endometrium or uterine contraction to expel the device [1, 6, 7, 10].

Though our case was visiting several health facilities with pelvic complaints, the diagnosis of retained IUD was not made until her current visit to this specialized hospital. In general, when patients report a history of IUD use and pelvic symptoms, diagnosis of retained IUD is usually established by speculum and pelvic US examination [4, 13]. Transvaginal ultrasound examination is superior to pelvic examination to confirm the location of IUD [14]. But in our case, the radiologist did not confirm the presence of the device in the uterus. Diagnosis of dislocated or migrated IUD requires an abdominopelvic X-ray, computerized tomography (CT) scan, or magnetic resonance imaging [MRI] [5, 6, 10]. In our case, the diagnosis was made by speculum examination.

IUD can be retained or forgotten in the uterus or outside the uterus for several reasons ranging from 22 to 50 years [1, 4, 14]. One of the main reasons why IUDs were forgotten in the uterus more than the expected time is poor counseling during insertion of the device and documentation [4, 9]. Our case was not adequately counseled and informed what to do with the device.

It is not easy to remove Lippes loop IUDs retained for several years [7, 14]. Lippes loop IUDs tend to accumulate small deposits of calcium causing corrosion in the plastic. This compromises the strength of the device and tail rendering it liable to fracture & breakage [15, 16]. In addition, these retained loops, tend to bury in the endometrium resulting in difficult removal with accompanying pain & bleeding. Removal may become more difficult after menopause because of atrophy of the uterus & cervical canal [4]. Because of these reasons, little literature recommends ripening the cervix with misoprostol [15, 16]. Some difficult cases require laparotomy or laparoscopy [5, 17]. In our case,

## Conclusion

Different works of the literature showed that retained Lippes loop IUD carry some complications. Our case had also postmenopausal pelvic pain. Therefore, we recommend the removal of IUDs at their expiry date or menopause.

## Data Availability

The datasets used during the current study are available from the corresponding author on reasonable request.

## Abbreviations

CT: Computerized tomography
IUD: Intrauterine device
MRI: Magnetic resonance imaging
PO: Per os
WHO: World Health Organization
